# Hotspots and status of Fetal Alpha-Thalassemia from 2009 to 2023: a bibliometric analysis

**DOI:** 10.3389/fped.2024.1467760

**Published:** 2024-12-11

**Authors:** Qiuying Li, Xinyan Li, Sheng He, Jiao Li

**Affiliations:** ^1^ Department of Ultrasonography, Maternity and Child Health Care of Guangxi Zhuang Autonomous Region, Nanning, China; ^2^ Graduate School, Guangxi University of Chinese Medicine, Nanning, China; ^3^ Birth Defects Prevention and Control Institute of Guangxi Zhuang Autonomous Region, Nanning, China; ^4^ Maternity and Child Health Care of Guangxi Zhuang Autonomous Region, Nanning, China

**Keywords:** α-thalassemia, bibliometrics, visual analysis, Citespace, VOSviewer

## Abstract

**Objective:**

to evaluate the research status and development hotspots of fetal α-thalassemia by quantitatively analyzing the diagnostic status, key areas, related management measures and prospects of the disease by bibliometrics.

**Methods:**

The global literature on fetal α-thalassemia and severe α-thalassemia from 2009–2023 in the Web of Science Core Collection (WOSCC) was visually analyzed by VOSviewer and CiteSpace.

**Results:**

(1) The examination of the quantity of publications concerning fetal α-thalassemia indicates a rising tendency prior to 2018, followed by a decrease after 2018. (2)The United States, China, Italy, Thailand have published more papers, and the United States has more collaborating countries such as Italy and China. (3) Chiang Mai University and Harvard University are the top two institutions with the highest contribution. However, Chiang Mai University's H index (12) and citation frequency per article (8.05) are relatively low and the NC (6,342), H index (33) and citations per article (75.42) of Harvard University are higher than those of the other institutions. (4) Tongsong T, Gambari R and Fucharoen S are the top three prolific authors. Fucharoen S emerges as the most frequently cited author with 738 citations, excluding self-citations. (5) HEMOGLOBIN leading with 87 published papers (NC:601,IF: 0.82, H-index: 13), followed by BLOOD(58 papers, Nc: 3755, IF: 25.48, H-index: 40) and BLOOD CELLS MOLECULES AND DISEASES(39 papers, Nc: 729, IF: 2.37, H-index: 16). (6) The most cited article was published in science and the second and third cited articles were featured in the Proceedings of the National Academy of Sciences; the top 3 clusters of co-cited literature are “gene editing”, “polymorphisms”, “hydroxyurea”. (7) Keywords analysis showe that the top two categories of keyword cluster focus on the prenatal diagnosis and the current treatment strategy of the disease, which remain the research hotspots.

**Conclusions:**

Recent research on this topic has primarily focused on prenatal diagnosis and treatment strategies. A particular area of interest is the ongoing research on gene therapy.The advances in non-invasive diagnosis and therapeutic methods will change the current management approaches for fetal severe α-thalassemia in the future.

## 1 Introduction

Alpha-thalassemia(α-thalassemia) is a notable issue in global health (1). Severe α-thalassemia, also called Hemoglobin Bart's hydrops fetalis syndrome, is a hemoglobinopathy resulted from inactivating or deleting all four α-globin alleles. This syndrome is commonly found in Southeast Asia and is characterized by a high incidence of fetal edema. Hemoglobin Bart's (*γ*4, Hb Bart's) has an unusually strong affinity for oxygen and cannot effectively deliver oxygen to fetal tissue (2, 3). The fetus may present with hypoxia, anemia, edema, death, or stillbirth. Furthermore, continued pregnancy increases the risk of severe complications in pregnant women, including preeclampsia, dystocia, and postpartum hemorrhage, and others (4). The prognosis of the disease is unfavorable, and the current clinical approach involves early prenatal diagnosis and induction of labor. Quantitative analysis of the status quo, critical aspects, associated management strategies, and prospects of fetal α-thalassemia holds substantial importance for efficient management.

Bibliometrics is a method of quantifying and evaluating the numbers and contents in a publication (5, 6). The bibliometric index include numbers of publications (Np), numbers of citations (Nc) which serve as measures of the annual global citation frequency of a publication, the H-index which combines publication frequency and citation frequency to create a commonly used influence index (7, 8), and the impact factor (IF) which is a valuable tool for assessing the influence and quality of journals. Based on the analysis of the characteristics of data and literature, this paper evaluates and predicts the development trend and hot research direction in the research field, guides the experimental strategy and fund decision-making, and provides effective evidence (9). Presently, bibliometric methods have been utilized to summarize the research on low-intensity ultrasound, breast cancer radiotherapy, polycystic ovary syndrome (10–12), and so on. However, bibliometric studies on α-thalassemia have not yet been carried out. The aim of this study was to explore the researches on fetal α-thalassemia, and to evaluate the research status and development hotspots in this field.

## 2 Materials and methods

In this research, the Web of Science Core Collection (WOSCC) with Citation Index (CI) was utilized for literature retrieval. To mitigate potential bias stemming from the rapid database updates, literature retrieval was performed on the same day to capture the comprehensive global literature on fetal α-thalassemia and severe α-thalassemia types spanning from January 2009 to December 2023, encompassing a 15-year period. The search strategy employed was as follows: TS = (fetal A thalassemia) OR TS = (fetal alpha thalassemia) OR TS = (fetal Hb Bart's Disease) OR TS = (fetal hemoglobin Bart's Disease). The data was exported as a plain text file, with CiteSpace utilized for time span clustering analysis and literature citation burst analysis. The Java program VOS viewer was employed for literature co-citation analysis, keyword co-occurrence network, and author cooperation analysis. When using VOS viewer analysis, we set the minimum number of documents or citations of each analysis content, so that the threshold reaches the maximum value less than 200.

## 3 Results

### 3.1 Trend of annual publication number of fetal α-thalassemia

**Figure 1:**
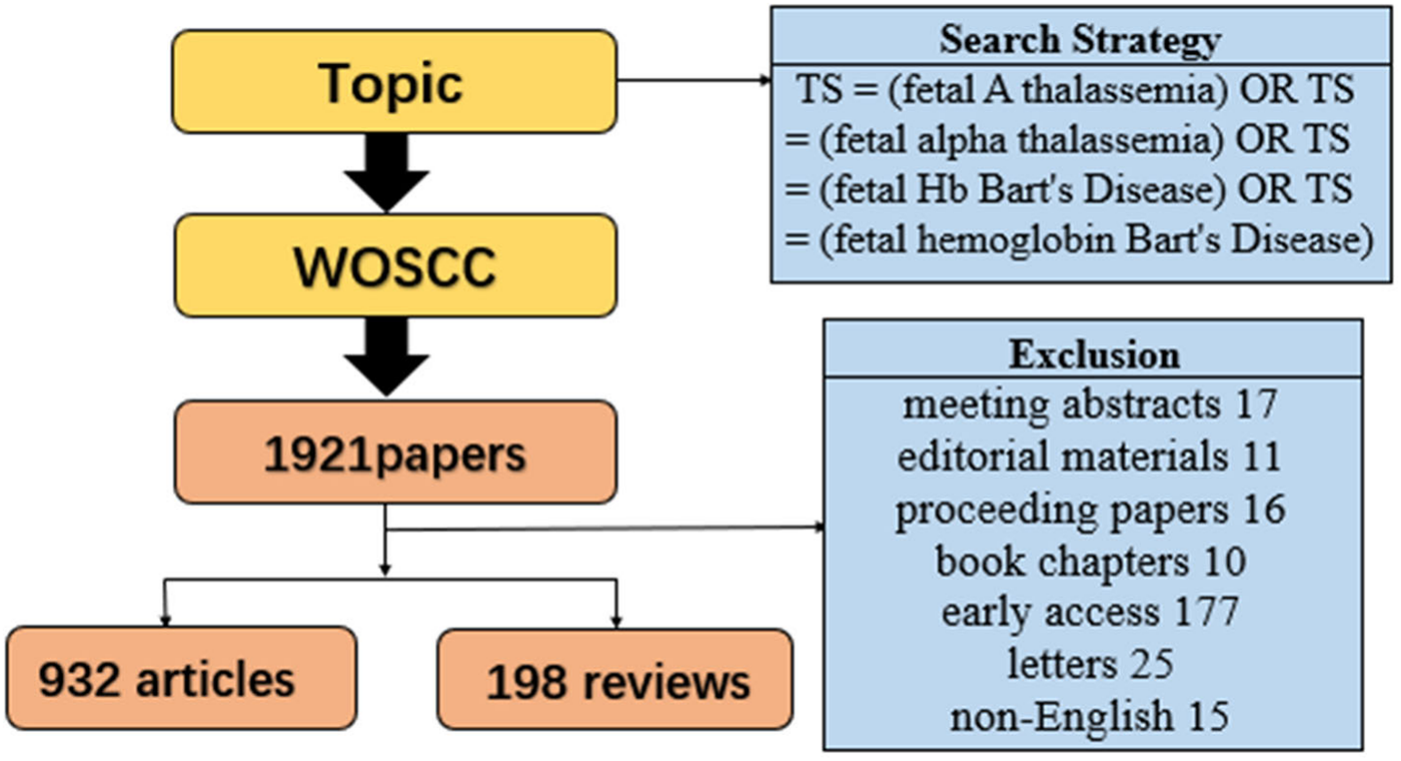
Flow chart for screening included literature.

**Figure 2:**
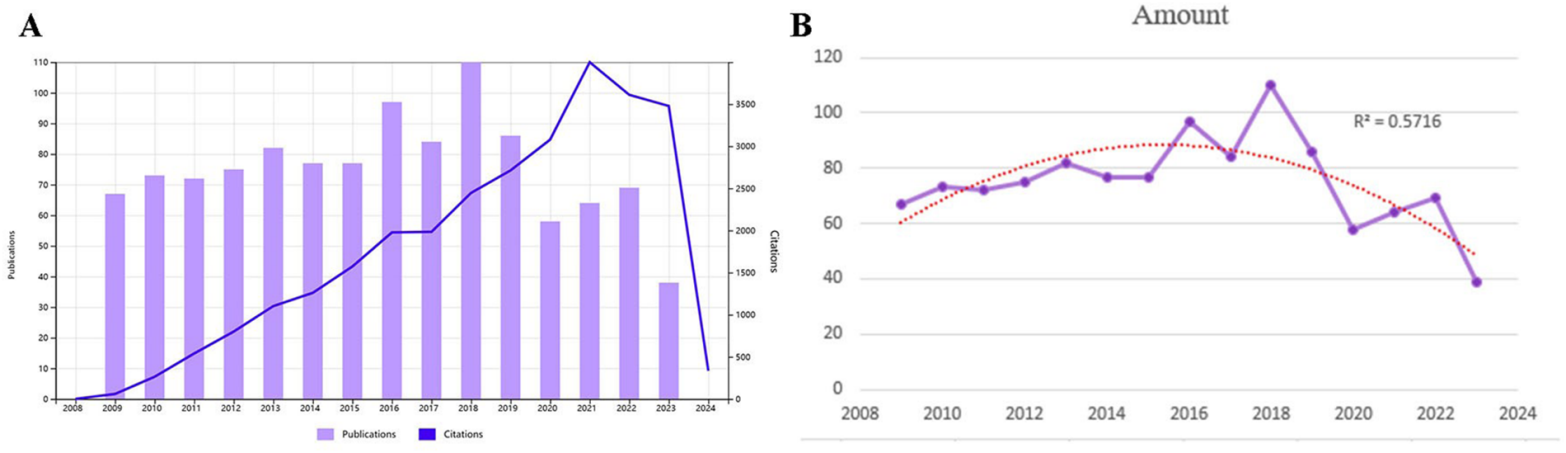
Publications and citations of each year in the past 15 years **(A)**. Fits the relationship curve between Np and publication year (R2 = 0.5716) **(B)**.

The final screening of 1,130 valid literatures included only works and reviews written in English from the retrieved publications. The detailed process is illustrated in Figure 1. Figure 2A depicts the variation in the Np pertaining to fetal α-thalassemia and the change curve of citation frequency over the course of the last 15 years. The examination of the quantity of publications concerning fetal α-thalassemia indicates a rising tendency prior to 2018, followed by a decrease after 2018. The number of publications escalated from 67 in 2009 to 110 in 2018, subsequently dwindling to 39 in 2023. The citation frequency continued to rise, peaked in 2021, and then showed a slight downward trend. The correlation coefficient between the annual quantity of publications and the multiple fitting curve of the publication year is 0.5716, as demonstrated in Figure 2B.

### 3.2 Trend analysis of national and regional publications

**Figure 3:**
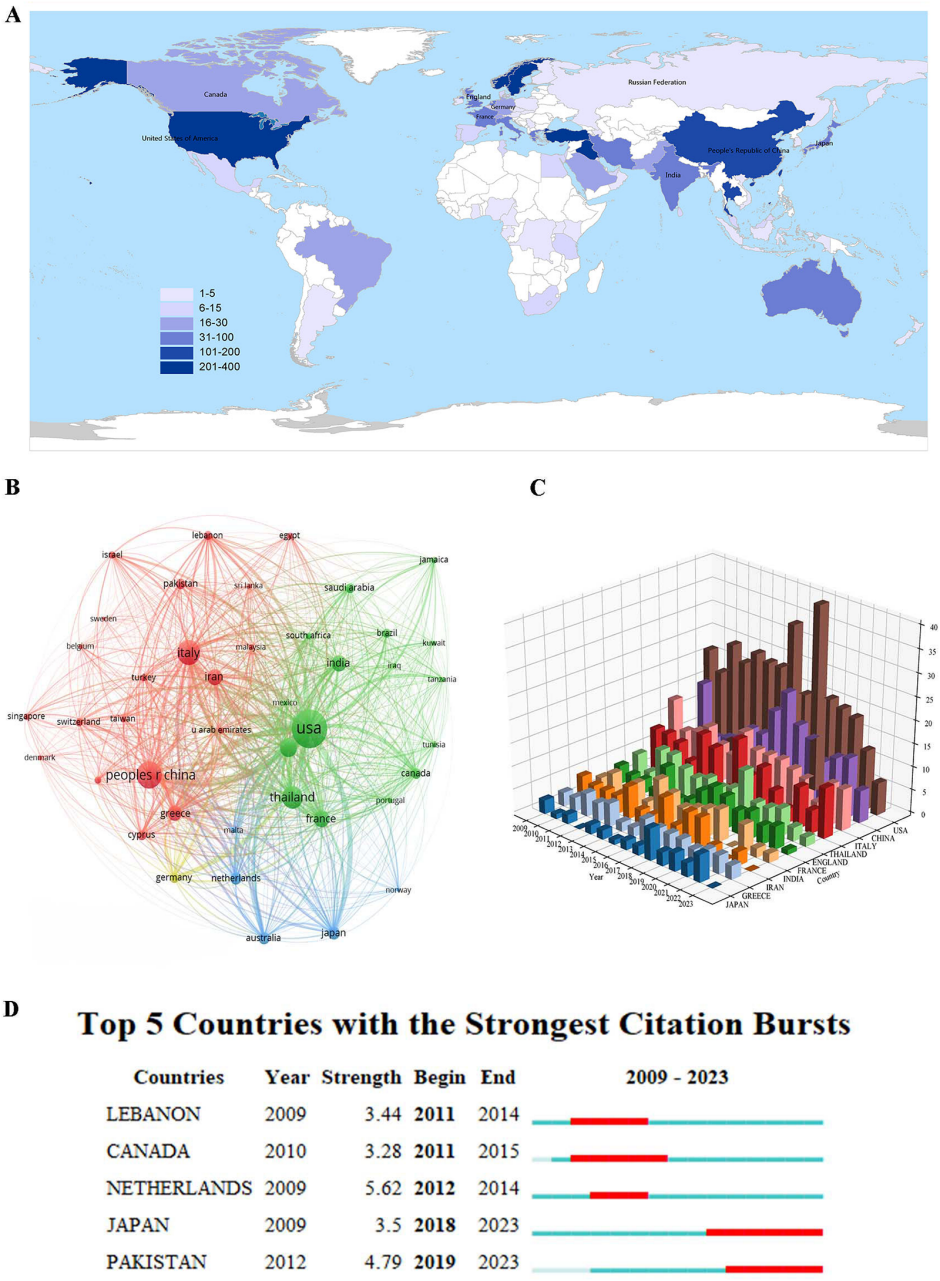
Global geographical distribution map of countries in the field of fetal α-thalassemia research **(A)**. National co-citation analysis map of fetal α-thalassemia research **(B)**. A 3D histogram of the number of papers published by countries in the study of fetal alpha-thalassemia in the past 15 years **(C)**. Analysis map of national emergent value in the field of α-thalassemia research **(D)**.

A comprehensive review of the literature indicates that 69 countries have contributed articles in this field between 2009 and 2023. The United States emerged as the leading contributor with 334 papers, representing 29.56% of all publications; the following countries are China, Italy, Thailand, and others, as shown in Table 1. Figure 3A shows the regional distribution of articles published in this field. The combination of Figure 3A,B illustrates the correlation between the quantity of publications and the size of nodes, as well as the extensive level of collaboration among nations. Notably, the United States, China, and Italy are represented by larger nodes, with the United States demonstrating a higher overall level of connectivity. Furthermore, the United States engages in collaborative efforts with 46 countries, including particularly close partnerships with Italy, China, and the United Kingdom. Figure 3C shows the changes in the annual number of documents issued by each country intuitively. A citation bursts analysis of Citespace reveals that Pakistan has experienced the most rapid growth in terms of publications since 2018 and is the country with the most significant increase in influence. However, the Netherlands is the country with the highest incidence of sudden citation, which was in an outbreak state from 2012–2014, as presented in Figure 3D.

**Table 1:** The top 10 countries with the highest number of publications in the field of fetal α-thalassemia research.

Rank	Country	NP	NC	H-index	Average citation per item	Percentage of NP(1,130)
1	USA	334	14,200	67	46.07	29.56%
2	CHINA	175	3,252	30	19.97	15.49%
3	ITALY	139	5,635	34	43.17	12.30%
4	THAILAND	120	1,532	21	14.49	10.62%
5	ENGLAND	83	4,761	31	58.49	7.35%
6	FRANCE	67	3,063	26	46.81	5.93%
7	INDIA	63	714	15	12.11	5.58%
8	IRAN	60	750	17	13.35	5.31%
9	GREECE	55	2,119	17	40.44	4.87%
10	JAPAN	38	1,979	20	53.63	3.36%

Abbreviations: NP, number of publications; NC, number of citations.

### 3.3 Analysis of affiliation relationship

**Figure 4:**
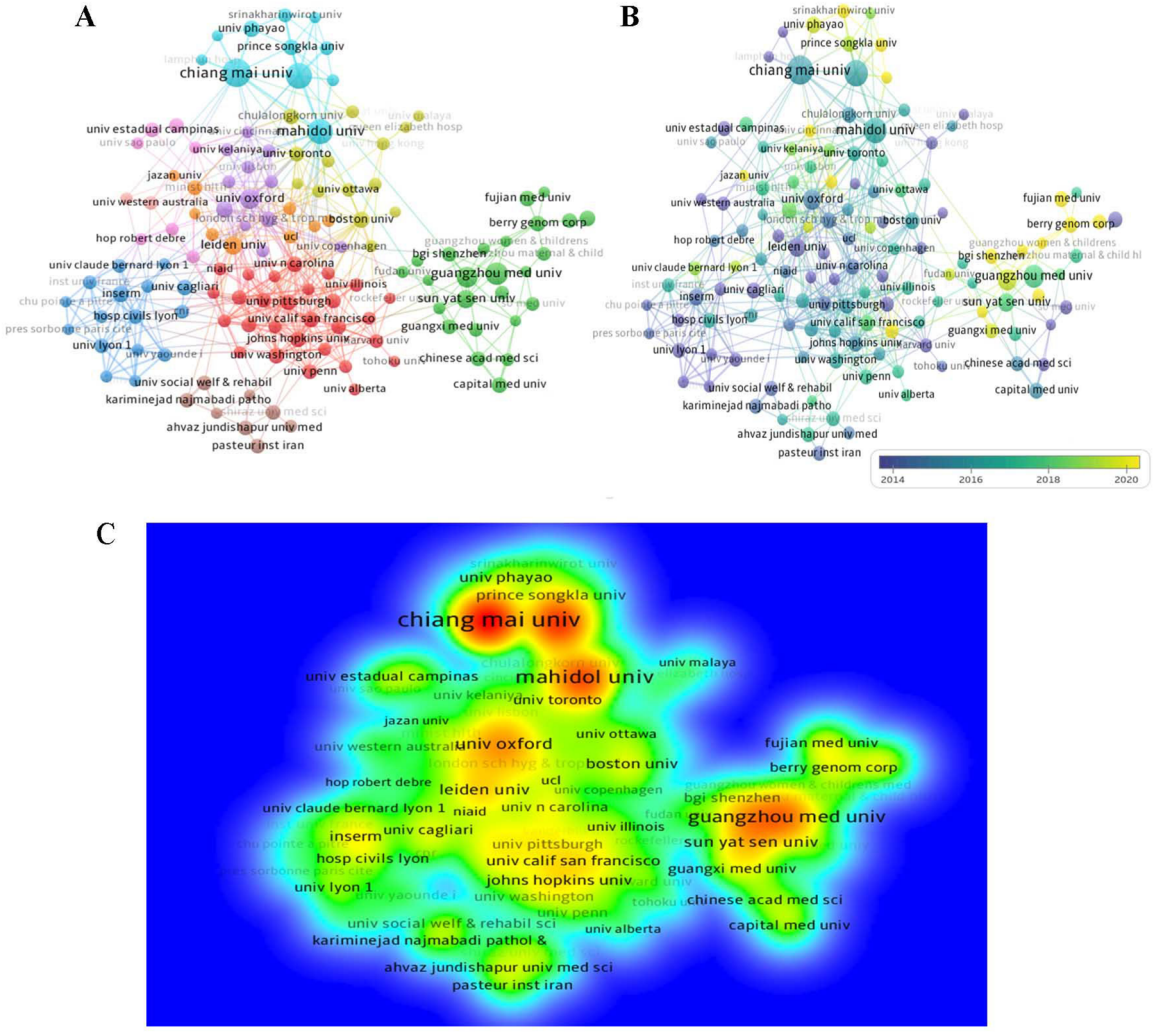
Co-occurrence analysis map of publishing institutions **(A)**. Time map of co-occurrence analysis of publishing institutions **(B)**. Density map of co-occurrence analysis of publishing institutions **(C)**.

The top 10 institutions were summarized according to the statistics of the affiliated institutions of the published literature related to fetal α-thalassemia in the past 15 years (Table 2). Among the institutions, Chiang Mai University in Thailand stands out as having published the most researches (55). However, its H index (12) and citation frequency per article (8.05) are relatively low. The institutions with the most papers were predominantly from the USA (70%, 7/10). The NC (6,342), H index (33), and citations per article (75.42) of Harvard University are relatively higher than those of the other institutions. The collaboration among institutions is characterized by a close relationship, delineated into nine clusters as depicted in Figure 4A. When considering the temporal distribution of institutional publications (Figure 4B) and the frequency heat map of institutional publications (Figure 4C), it is evident that CHIANG MAI UNIVERSITY and MAHIDOL UNIVERSITY in Thailand initiated research activities at an earlier stage, as indicated by a concentrated red area on the heat map. In contrast, GUANGZHOU MEDICAL UNIVERSITY in China commenced research activities at a later stage, also reflected by a concentrated red area on the heat map.

**Table 2:** The top 10 institutions with the highest number of publications in the field of fetal α-thalassemia research.

Rank	Affiliations	Country	NP	NC	H-index	Average citation per item
1	CHIANG MAI UNIVERSITY	Thailand	55	342	12	8.05
2	HARVARD UNIVERSITY	USA	50	3,642	33	75.42
3	UNIVERSITY OF FERRARA	Italy	48	624	17	15.75
4	UNIVERSITY OF LONDON	USA	45	2,489	24	56.44
6	HARVARD MEDICAL SCHOOL	USA	40	2,985	28	77.2
5	INSTITUT NATIONAL DE LA SANTE ET DE LA RECHERCHE MEDICALE INSERM	France	39	1,531	21	40.1
7	BOSTON UNIVERSITY	USA	37	2,645	21	73.49
8	DANA FARBER CANCER INSTITUTE	USA	37	2,997	27	83.43
9	NATIONAL INSTITUTES OF HEALTH NIH USA	USA	37	1,771	21	48.3
10	BOSTON CHILDREN S HOSPITAL	USA	36	2,839	27	81.47

Abbreviations: NP, number of publications; NC, number of citations.

### 3.4 Analysis of authors of published literature

**Figure 5:**
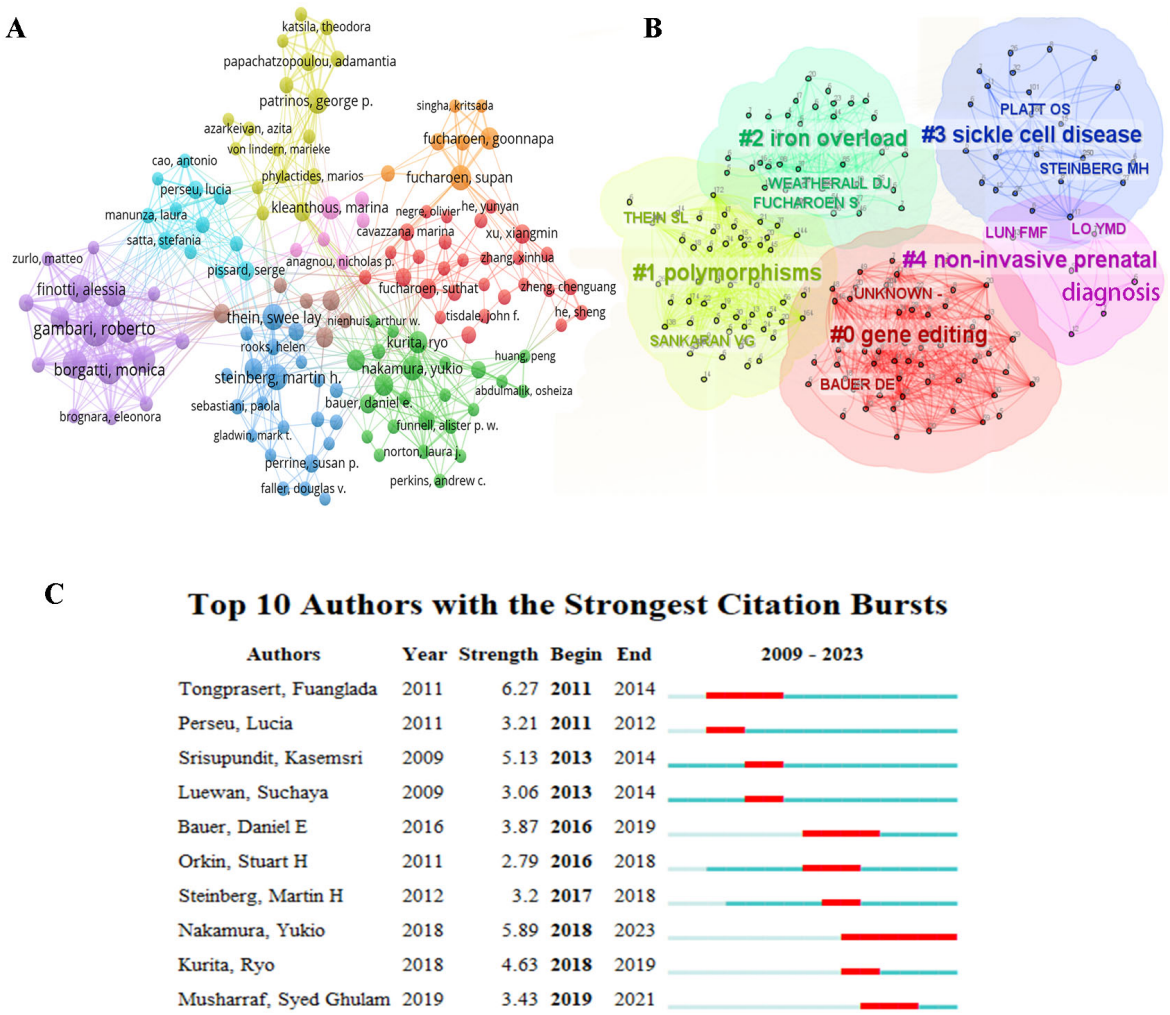
Co-citation analysis map of authors in the field of fetal α-thalassemia research. **(A)** Cluster diagram of cited keywords of authors **(B)** The author's emergent value analysis map in the field of fetal α-thalassemia research **(C)**.

The data presented in Table 3 reveals the top ten authors with the highest Np in the field over the last 15 years. Tongsong T, Gambari R and Fucharoen S are the top three prolific authors. Fucharoen S emerges as the most frequently cited author with 738 citations, excluding self-citations. The following closely are Gambari R and Borgatti M, with 534 and 525 citations respectively. As depicted in Figure 5A,B, the author and the author's cited visual analysis of keyword clustering suggest that these individuals serve as central figures within their respective organizations. Moreover, their academic pursuits in the field began in the years 2014, 2015, and 2015 respectively, with the most intense correlation between their nodes, suggesting a higher level of collaboration and highlighting their crucial role in the research of fetal α-thalassemia. Among the top 10 authors with the most citation bursts, Tongprasert F, Nakamura Y, and Srisupundit K are the most prominent, with Nakamura, Y experiencing a notable increase in citations since 2018, as illustrated in Figure 5C.

**Table 3:** The top 10 authors with the highest number of publications in the field of fetal α-thalassemia research.

Rank	Author	Affiliations	Country	NP	NC	H-index
1	Tongsong T	Chiang Mai University	Thailand	45	258	12
2	Gambari R	Ferrara University	Italy	43	534	16
3	Fucharoen S	Khon Kaen University	Thailand	35	738	16
4	Luewan S	Chiang Mai University	Thailand	33	255	10
5	Srisupundit K	Chiang Mai University	Thailand	33	239	9
6	Borgatti M	Ferrara University	Italy	31	525	14
7	Tongprasert F	Chiang Mai University	Thailand	31	197	7
8	Zuccato C	Ferrara University	Italy	28	420	13
9	Finotti A	Ferrara University	Italy	26	348	13
10	Breveglieri G	Ferrara University	Italy	25	345	13

Abbreviations: NP, number of publications; NC, number of citations.

### 3.5 Published journal analysis

**Figure 6:**
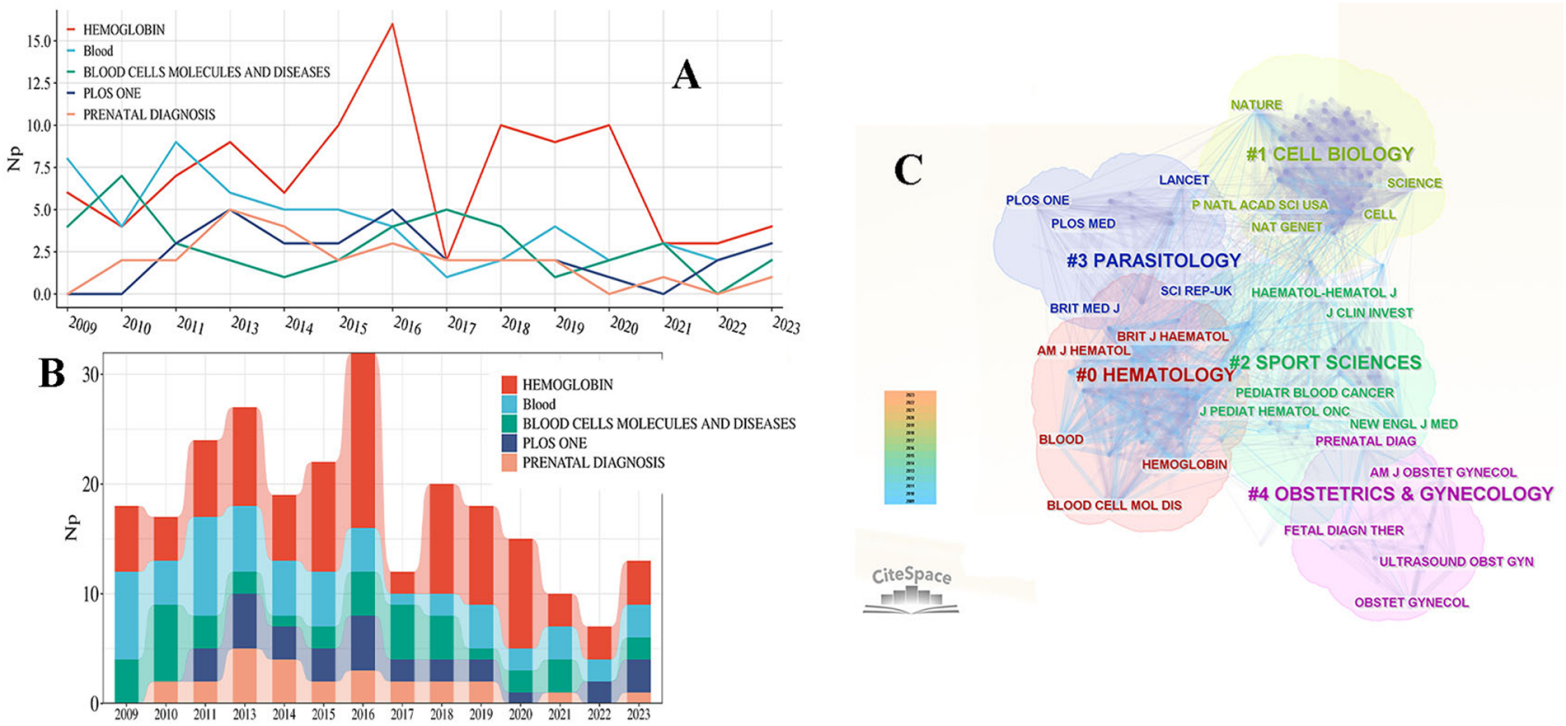
The annual growth curve of the number of publications of the top 5 journals **(A)**. The annual publication volume stack diagram of the top 5 journals **(B)**. Journal subject category clustering diagram of published articles **(C)**.

This field has been covered in 200 journals over the past 15 years. The analysis focused on the ten most productive journals (Table 4), with HEMOGLOBIN leading with 87 published papers (IF: 0.82, H-index: 13), followed by BLOOD and BLOOD CELLS MOLECULES AND DISEASES. However, BLOOD displayed the highest non-self-cited literature citation frequency (Nc: 3755, IF: 25.48, H-index: 40), and average number of citations (66.09). Following closely behind was the BRITISH JOURNAL OF HAEMATOLOGY. The data presented in Figure 6A,B show the changes in the annual Np of the top five journals. Specifically, HEMOGLOBIN stands out with a significantly higher Np compared to other top five journals in 2016. During the period from 2011–2016, most of the journals experienced a peak in publications, suggesting a heightened focus on the respective fields. The primary subjects covered by these journals include hematology, cell biology, sports science, parasitology, and obstetrics and gynecology. Figure 6C illustrates the clustering of journals based on subject matter, providing researchers with guidance on selecting appropriate publications for their work.

**Table 4:** Top 10 journals in the field of fetal α-thalassemia research.

Rank	Journal	NP	NC	IF(2022)	H-index	Average citation per item
1	HEMOGLOBIN	87	601	0.82	13	7.14
2	BLOOD	58	3,755	25.48	40	66.09
3	BLOOD CELLS MOLECULES AND DISEASES	39	729	2.37	16	19.1
4	PLOS ONE	32	640	3.75	13	20.13
5	PRENATAL DIAGNOSIS	28	498	3.24	13	18.21
6	BRITISH JOURNAL OF HAEMATOLOGY	24	812	8.62	16	34.08
7	SCIENTIFIC REPORTS	24	162	4.99	9	6.79
8	JOURNAL OF PEDIATRIC HEMATOLOGY ONCOLOGY	19	157	1.17	7	8.47
9	ANNALS OF HEMATOLOGY	16	227	4.03	9	14.69
10	INTERNATIONAL JOURNAL OF MOLECULAR SCIENCES	16	92	6.21	5	5.81

Abbreviations: NP, number of publications; NC, number of citations; IF, impact factor.

### 3.6 Literature co-citation cluster analysis

**Figure 7:**
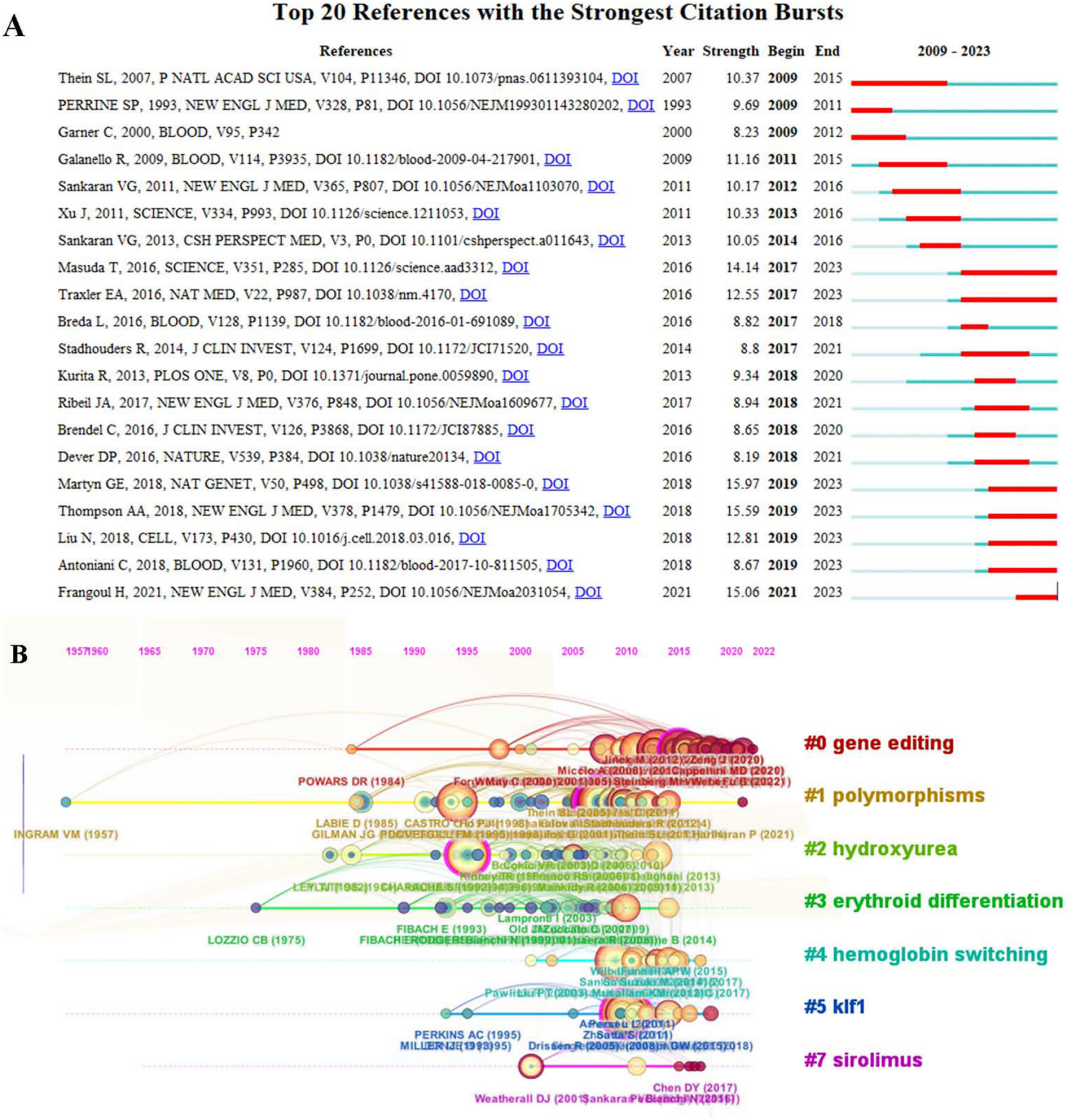
Representative top 20 papers with the strongest burst citations **(A)**. Co-cited literature keyword clustering visualization map **(B)**.

An analysis of the referenced literature spanning the last 15 years indicates that the majority of the top ten cited articles were published between 2007 and 2010, as shown in Table 5. The foremost article (13), published in Science, explores the role of BCL11A as a potential regulator of HbF expression, offering a new target for the treatment of thalassemia. It's primary role is to stimulate HbF reactivation and elevate fetal hemoglobin (HbF) levels through modulation of F cell production. The second and third most cited articles were featured in the Proceedings of the National Academy of Sciences (PNAS) (14, 15). The citation burst observed in Figure 7A highlights the scholarly works that are of particular interest to researchers during the specified period. Article (23) published in Nature Genetics has experienced a notable surge in influence within the field since 2019, characterized by a period of exponential growth. Figure 7B shows that the top 7 clusters of co-cited literature are “gene editing”, “polymorphisms”, “hydroxyurea”, “erythroid differentiation”, “hemoglobin switching”, “klf1”, “sirolimus”.

**Table 5:** Citation analysis of highly cited literature in the field of fetal α-thalassemia research.

Rank	First author	Article	Journal	Year of publication	Number of citations
1	Vijay G. Sankaran	Human Fetal Hemoglobin Expression Is Regulated by the Developmental Stage-Specific Repressor BCL11A (13)	Science	2008	209
2	Manuela Uda	Genome-wide association study shows BCL11A associated with persistent fetal hemoglobin and Amelioration of the phenotype of β-thalassemia (14)	Proc Natl Acad Sci USA	2008	177
3	Lettre, Guillaume	DNA polymorphisms at the BCL11A, HBS1l-MYB, and β-globin loci associate with fetal hemoglobin levels and pain crises in sickle cell disease (15)	Proc Natl Acad Sci USA	2008	153
4	Stephan Menzel	A QTL influencing F cell production maps to a gene encoding a zinc-finger protein on chromosome 2p15 (16)	Nature Genetics	2007	152
5	Borg, Joseph	Haploinsufficiency for the erythroid transcription factor KLF1 causes hereditary persistence of fetal hemoglobin (17)	Nature Genetics	2010	129
6	Platt, OS	Mortality In Sickle Cell Disease—Life Expectancy and Risk Factors for Early Death (18)	The New England Journal of Medicine	1994	116
7	Zhou, Dewang	KLF1 regulates BCL11A expression and γ- to β-globin gene switching (19)	Nature Genetics	2010	111
8	Sankaran	Developmental and species-divergent globin switching are driven by BCL11A (20)	Nature	2009	105
9	Thein, Swee Lay	Intergenic variants of HBS1l-MYB are responsible for a major quantitative trait focus on chromosome 6q23 influencing fetal hemoglobin levels in adults (21)	Proc Natl Acad Sci USA	2007	98
10	Bauer	An Erythroid Enhancer of BCL11A Subject to Genetic Variation Determines Fetal Hemoglobin Level (22)	Science	2013	92

### 3.7 Keyword cluster analysis and time series analysis

**Figure 8:**
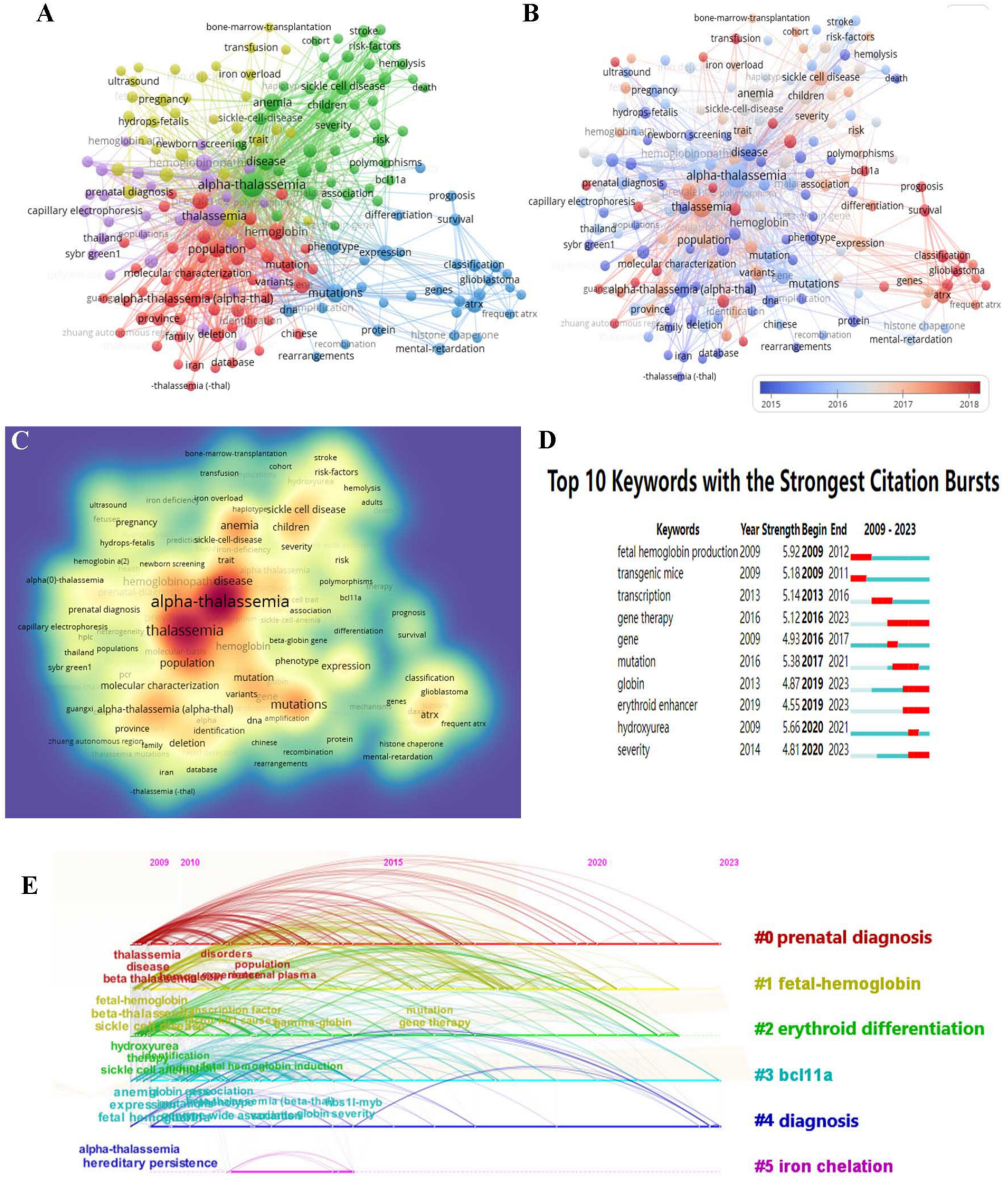
Keyword analysis map of α-thalassemia in fetus **(A)**. Keyword visualization based on APY **(B)**. Heatmap of keywords according to the average frequency of occurrence **(C)**. Top 10 keywords with the most vigorous citation bursts **(D)** timeline distribution of keyword clustering in the field of fetal α-thalassemia research **(E)**.

A keyword cluster analysis and a time series analysis were conducted on the frequency of citation of keywords in the field of fetal α-thalassemia research spanning the last 15 years. The analysis, presented in Table 6 and Figure 8A, identified the top ten most cited keywords as “fetal hemoglobin”, “sickle cell disease”, “transcription”, “gene therapy”, “expression”, “α-thalassemia”, “hydroxyurea”, “anemia”, “prevalence”, and “severity”. In Figure 8B, keywords categorized by their average publication year (APY), with red indicating more recent keywords from 2018 such as “classification”, “prognosis”, and “survival”. The heat map in Figure 8C illustrates the average frequency of keywords, with red indicating keywords that appear more frequently in the literature. Based on the examination of the top ten keywords (Figure 8D), it was determined that “fetal hemoglobin production” exhibited the highest intensity of outbreak, while “gene therapy” demonstrated the longest duration of outbreak until the year 2023. Additionally, the keyword co-citation time series analysis map (Figure 8E) indicated that cluster 0, focusing on prenatal diagnosis, is currently the primary area of research interest. Cluster 1 pertains to fetal hemoglobin, cluster 2 to erythrocyte differentiation, cluster 3 to bcl11a, cluster 4 to diagnosis, and cluster 5 to iron chelation. Combined with the above analysis, the predominant studies have focused on this domain, encompassing maternal plasma composition, gene therapy, erythrocyte differentiation, genetic modifiers, case reports, and current treatment strategies. In addition, researchers are encouraged to investigate potential treatment strategies for fetal α-thalassemia through the utilization of animal experiments.

**Table 6:** The top 10 keywords with the highest number of publications in the field of fetal α-thalassemia research.

Rank	keywords	Citation counts
1	Alpha-thalassemia	854
2	Thalassemia	398
3	Mutations	223
4	Disease	196
5	Anemia	194
6	Diagnosis	182
7	Prevalence	140
8	Population	137
9	Hemoglobinopathies	131
10	Hemoglobin	120

## 4 Discussion

This study presents a summary of the current research status of fetal α-thalassemia integrating bibliometrics and visual analysis. It delineates the emerging trends and identifies the most promising research avenues in this field. A quantitative analysis of global publication volume reveals a notable increase in the number of publications prior to 2018. This growth in annual publication volume is inextricably linked to the advancement of basic research and clinical trials. Based on the analysis of publications by national researchers, the countries such as the United States, China, Italy, and Thailand, where the most publications were published are largely congruent with the distribution of thalassemia diseases, were concentrated in tropical and subtropical regions. The United States exhibits the highest H-index (67) and Nc values (14,200). The majority of the top ten institutions with the most papers were predominantly from the USA, particularly HARVARD UNIVERSITY, suggesting the country's significant depth of academic research and strong scholarly capabilities in this field. Furthermore, the United States' extensive collaborative partnerships with other countries contribute to its elevated H-index and overall scholarly influence. Thailand is a country with a high prevalence of α-thalassemia and has the most published institutions CHIANG MAI UNIVERSITY and outstanding professional researchers, such as Tongsong T, Fucharoen S and Luewan S. However, its H index and Nc are low, and attention should be paid to improving the cooperation among countries. In this field, China stands out as one of the leading contributors, with numerous papers and extensive research activities, and GUANGZHOU MEDICAL UNIVERSITY being a recent contributor. Despite the large volume of publications in a brief period of time, particularly in the field of genetic molecular science, the H-index and Nc metrics of China are lower than those of Italy, which Np are lower than China. It suggests that the quality and impact of Chinese research outputs require enhancement (24, 25).

Among the journals with numerous publications, nine have higher impact factors (greater than 1). With the most articles published, HEMOGLOBIN has a lower IF (0.82), while the second-ranked BLOOD has the highest IF (25.48) and H index (40). The influences of these journals are so high, that it suggests that researchers interested in this field should pay attention to these journals and relevant authors. By examining the pertinent literature published in the journal, it is evident that BLOOD and HEMOGLOBIN are publications associated with hematology, while CELL and P NATL ACAD SCI USA are journals focused on cell biology, and PRENATAL DIAG is a journal specializing in obstetrics and gynecology. Therefore, scholars could contribute to their respective scientific disciplines, engage in discussions, and share knowledge with fellow academics.

The co-cited authors are categorized into 9 distinct clusters, allowing researchers to identify authors whose work aligns with their own research focus through the network of connections. By integrating the keyword clustering of cited authors, researchers can identify authors specializing in specific research areas, such as Bauer in the realm of gene editing, LUN F in the realm of non-invasive prenatal diagnosis. Although Fucharoen S may not have the highest number of published papers, his H-index (16) and Nc (738) are the highest in their field, establishing him as a scientist of significant influence. Similarly, Nakamura Y stands out for having the highest citation intensity and longest duration since 2018. By examining the achievements of these authors, researchers can gain valuable insights to develop new ideas and methods, ultimately fostering innovation in the field. The literature co-citation cluster analysis reveals that most highly cited papers (60%) pertained to the fundamental research of BCL11 A. Furthermore, the top ten articles with the strongest citation burst were published after 2016, indicating that the related topics have received considerable attention in recent years. It is noteworthy that the majority of these publications pertain to the expression of genes that affect fetal hemoglobin (23, 26–27).

Keyword clustering analysis is the key content of this paper. The first category of keyword cluster analysis focuses on prenatal diagnosis of the disease, which appears earliest and remains a hotspot for research. In the current system, pregnant women are tested for their hemoglobin levels and their red blood cell levels. If positive, further genetic testing is performed. If they are diagnosed as thalassemia gene carriers, their spouses should also take this exam. If both are carriers of the same type of thalassemia gene, they are high-risk couples, that is, their offspring may have severe type of thalassemia. Then DNA detection technology is necessary to identify fetal genotypes and gene mutation types (28–30). Srisupundit K has authored numerous scholarly articles in the discipline of hematology (31–33). Invasive methods such as villus biopsy, amniocentesis and umbilical cord puncture are used for sampling and then testing (34). The primary method for molecular diagnostics is polymerase chain reaction (PCR) (35–38). Nevertheless, conventional molecular diagnostic methods are intricate, offer restricted probe coverage, and are time-consuming. China's contributions to this area have advanced the utilization of Next-Generation Sequencing (NGS) technology for genetic screening of thalassemia (39–41). Several studies have suggested that third-generation sequencing (TGS) technology offers a substantial enhancement in the detection rate of thalassemia genes with Mediterranean gene variation and new structural variation when compared to NGS technology (42, 43). In recent years, there has been a growing interest among scholars in the advancement of high-throughput sequencing technology, which has facilitated quicker and more comprehensive diagnosis of thalassemia.

Non-invasive prenatal diagnosis (NIPD) encompasses noninvasive genetic diagnosis, noninvasive ultrasound prenatal diagnosis and maternal serological indicators. The non-invasive genetic diagnosis technology detects thalassemia genes by analyzing fetal nucleated red blood cells (FNR-BC) and cell-free fetal DNA (cffDNA) in maternal blood (44–48). However, the accuracy of this technology is constrained by the intricate nature of the procedure and the scarcity of fetal cells in the peripheral blood of pregnant individuals, leading to its limited adoption in clinical settings. Since the signs of increased fetal blood flow, enlarged heart, thickened placenta appear in severe α-thalassemia fetuses can be observed by ultrasound before the onset of hydrops symptoms, these markers can be served as predicting markers for them in high-risk pregnancies; and it has been proven that this non-invasive ultrasound markers are both cost-effective and efficient (49–51). Fetal cardiothoracic ratio (CTR) has been recognized as a highly effective predictor, with a commonly utilized threshold range of 0.50–0.52 (52, 53). Researches conducted in China have demonstrated that the Z score of fetal heart size may serve as a novel and sensitive indicator for identifying severe α-thalassemia (54, 55). Another study also found that the Z value of fetal cardiac volume (CV) to predict this disease may be better than CTR (56). For maternal serological indicators, Tongprasert F and other researchers have identified maternal serum alpha fetoprotein (AFP), placental like growth factor (PlGF), inhibin-A, and other biomarkers as potentially effective in predicting fetal α-thalassemia major during the second trimester (57–60).

The second category of keyword clustering focuses on the current treatment strategy of the disease. Fetuses diagnosed with severe α-thalassemia generally need to terminate in time due to poor prognosis and high incidence of complications in pregnant women. With the progress of modern medicine, the possible treatment options include intrauterine transfusion (IUT) and postnatal bone marrow transplantation. While some rare cases have been effectively managed prenatally (61–63), some of them may experience complications such as chronic hypoxemia, postnatal respiratory failure, and pulmonary hypertension (63). Hematopoietic stem cell transplantation (HSCT) for severe α-thalassemia is practical option with a high cure rate (64, 65). However, due to factors such as rejection and donor sources, intrauterine HSC transplantation has not yet been successful (66). Choosing the appropriate HSC source, optimizing the scheme, and timing of intrauterine transplantation are still challenging. Another potentially promising therapy is gene therapy such as gene editing (67); virus vector; small RNA interference, etc, which is more promising to intervene in fetus. Constrained by donor availability and limited funding, current research and development efforts in gene therapy primarily concentrate on β-thalassemia (68). Although the progress of gene therapy for severe fetal α-thalassemia is slow, clinical trials are gradually being carried out (69). A studiy have found the possibility of applying protein replacement therapy to α-thalassemic (HBH) by PTD technology to recombine α-globin chains (70). Although there have not been any reported cases of fetal α-thalassemia treated with gene therapy to date, we believe that the advances in genetic technology may have the potential to revolutionize current management approaches for fetal severe α-thalassemia in the future (69, 71).

This study utilized bibliometric analysis to explore the theme of fetal α thalassemia (72), focusing solely on SCI-extended English articles and reviews. However, this approach may exclude recently published high-quality articles with limited citations, thereby introducing certain limitations. Citespace focuses on individual publication analysis and Vosviewer emphasizes cluster analysis (73), both software tools are unable to analyze the complete text of articles, potentially leading to information omission and delays.

## 5 Conclusion

Our findings suggest that research on fetal α-thalassemia has developed steadily, with a concentration of literature in papers. Fucharoen S is identified as the author with the highest citation frequency of non-self-cited literature. The United States leads in both publications and citations in this field, with HARVARD UNIVERSITY having the highest H index. The journal BLOOD stands out for its high citation frequency of non-self-cited literature. The primary areas of focus in fetal α-thalassemia research include prenatal diagnosis and disease management, epidemiological studies, genetic investigations, molecular biology and intervention, and current treatment modalities. A particular area of interest is the ongoing research on treatment strategies, with gene therapy representing current frontier research in the field. The advances in non-invasive diagnosis and therapeutic methods will change current management approaches for fetal severe α-thalassemia in the future.

## Data Availability

The original contributions presented in the study are included in the article/Supplementary Material, further inquiries can be directed to the corresponding author.
